# Phylogenetic comparative analysis: Chemical and biological features of caseins (alpha-S-1, alpha-S-2, beta- and kappa-) in domestic dairy animals

**DOI:** 10.3389/fvets.2022.952319

**Published:** 2022-09-15

**Authors:** Abdallah A. Hassanin, Ali Osman, Osama Osman Atallah, Mohamed T. El-Saadony, Sameh A. Abdelnour, Heba S. A. Taha, Mohamed F. Awad, Hany Elkashef, Ahmed Ezzat Ahmed, Ibrahim Abd El-Rahim, Abdullah Mohamed, Ahmed S. Eldomiaty

**Affiliations:** ^1^Genetics Department, Faculty of Agriculture, Zagazig University, Zagazig, Egypt; ^2^Department of Biochemistry, Faculty of Agriculture, Zagazig University, Zagazig, Egypt; ^3^Department of Plant Pathology, Faculty of Agriculture, Zagazig University, Zagazig, Egypt; ^4^Department of Agricultural Microbiology, Faculty of Agriculture, Zagazig University, Zagazig, Egypt; ^5^Animal Production Department, Faculty of Agriculture, Zagazig University, Zagazig, Egypt; ^6^Department of Biology, College of Science, Taif University, Taif, Saudi Arabia; ^7^Dairy Department, Faculty of Agriculture, Cairo University, Giza, Egypt; ^8^Department of Biology, College of Science, King Khalid University, Abha, Saudi Arabia; ^9^Department of Theriogenology, Faculty of Veterinary Medicine, South Valley University, Qena, Egypt; ^10^Department of Environmental and Health Research, Umm Al-Qura University, Mecca, Saudi Arabia; ^11^Infectious Diseases, Department of Animal Medicine, Faculty of Veterinary Medicine, Assiut University, Asyut, Egypt; ^12^Research Centre, Future University in Egypt, Cairo, Egypt

**Keywords:** casein protein, phylogenetic analysis, bioinformatics, antimicrobial, antioxidant

## Abstract

Caseins determine the physicochemical, physiological, and biological characteristics of milk. Four caseins—alpha-S-1, alpha-S-2, beta, and kappa—were analyzed phylogenetically and *in silico* and characterized regarding chemical, antimicrobial, and antioxidant features in five dairy animals: Arabian camels, sheep, goats, cattle, and water buffalos. The sequence of full-length amino acids of the four caseins for the five species was retracted from the NCBI GenBank database. Multiple sequence alignment is used to examine further the candidate sequences for phylogenetic analysis using Clustal X and NJ-Plot tools. The results revealed that sheep and goats possess strong similarities (98.06%) because of their common ancestor. The same was observed with cattle and water buffalos (96.25%). The Arabian camel was located in a single subclade due to low similarity in casein residues and compositions with other dairy animals. Protein modeling showed that alpha-S1- and alpha-S2-caseins possess the highest number of phosphoserine residues. The *in silico* computed chemical properties showed that β-casein recorded highest hydrophobicity index and lowest basic amino acid content, while α-S2-casein showed the opposite. The computed biological parameters revealed that α-S2-casein presented the highest bactericidal stretches. Only Arabian camel β-casein and k-casein showed one bactericidal stretches. The analysis also revealed that β-casein, particularly in Arabian camels, possesses the highest antioxidant activity index. These results support the importance of the bioinformatics resources to determine milk casein micelles' chemical and biological activities.

## Introduction

Casein in milk is widely consumed in the human diet worldwide, particularly in developing countries. Because of its widespread availability and enormous production quantities, cattle milk is the most consumed milk worldwide as a valued source of human nutrition. Non-bovine milk is essential to people in developing countries (1, 2). For example, buffalo milk in Asian countries, sheep milk in Europe and the Middle East, camel milk in Africa and some Asian countries, and goat milk in Africa and southern Asia (1, 3).

The majority of studies on milk casein have been conducted on cow milk as the most produced and consumed milk around the world (4); however, very few studies have been conducted on the comparison of milk from different domestic dairy animal species (5) and non-dairy animals, where these properties are explicit and may provide insight (6). The main nutritional benefit of milk is its protein content (of which around 80% is casein) (7). The casein proteins including four classes, namely, alpha S-1, alpha S-2, beta, and kappa, are assembled into a structure called casein micelles (8). Milk casein of different animal species possesses variations in amino acid sequences. The average size of casein micelles can also vary significantly from one species to another (9, 10). Animal species have variations in casein ratios (11) and micelle sizes (12). Caseins are almost similar in molecular weight in different domestic dairy species (13).

Comparative genomic analysis can provide new insights into the functionality of casein genes with respect to the caseins. Comparative genomic analysis is a rapidly emerging field in computational biology whereby two or more genomes are compared to obtain a global view of genomes and their deduced proteomes and assign previously unknown functions to genes with respect to their proteins (14).

Computational chemical properties can also pave the way to predicting the properties of deduced proteins. The calculated propensity index, which is deduced from the associated half-maximal inhibitory concentration (IC_50_) value for each amino acid alteration, serves as a useful benchmark for evaluating protein sequence determinants. Because low IC_50_ values imply greater antimicrobial activity, amino acids having a lower bactericidal propensity value (PV) are more likely to be used in antimicrobial peptides. Residues with positive charges (Arginine, Lysine, and Histidine) and some hydrophobic residues (Tryptophane, Tyrosine, and Valine) are unfavorable and present a low propensity index, whereas negatively charged residues are unflavored and show a high propensity index. Antimicrobial proteins would require positively charged residues to drive them to the negatively charged bacterial cell wall and cytoplasmic membrane, where they exert their antimicrobial effect (15). To form pores or other destabilizing structures that lead to membrane depolarization or local disruption and, finally, bacterial cell death, hydrophobic residues would need to interact with lipophilic regions of lipid bilayers (16). Interestingly, tryptophane (W) has the lowest PV value among the hydrophobic residues, whereas leucine (L) has the highest value, and isoleucine (I) and valine (V) are favored over L. Moreover, W residues are known to play a role in peptide antibacterial activity (17).

Several studies have shown that milk casein has hydrophilic, hydrophobic, antimicrobial, antioxidant, and anticancer properties (18–21). These properties offer great potential use of casein micelles as food additives in food industries instead of the use of synthetic additives, which cause some side effects: allergic, intoxications, cancer, and other degenerative diseases. The presence of phosphate groups near the peptide chain results in polar, acidic domains that are good for sequestering divalent metals, such as calcium, zinc, copper, manganese, and iron. An anionic triplet embedded in the bioactive peptide (SerP–SerP–SerP–Glu–Glu) is a distinguishing property of every functional CPP generated from whole and individual casein micelles (22, 23).

*In silico* analysis for caseins enables the accurate prediction of the computed chemical properties and antimicrobial, and antioxidant activities. Thus, this study was conducted *in silico* to determine the phylogenetic relationship, three-dimensional structure, chemical, and biological features of caseins in five domestic dairy animals: Arabian camels, sheep, goats, cattle, and water buffalos.

## Methodology

### Animals and sampling

The caseins—alpha-S1-, alpha-S2-, beta-, and kappa-casein—were comprehensively *in silico* and characterized in five milk-producing animals, namely Arabian camels, sheep, goats, cattle, and water buffalos, to determine their genetics, chemical, and biological features.

### Bioinformatics analysis of caseins

#### Phylogenetic analysis

Amino acid sequences for casein subunits—alpha-S1, alpha-S2, β-, and k-casein—were retracted from the NCBI (https://www.ncbi.nlm.nih.gov/genbank/) in FASTA format. The accession number of each protein is presented in Table 1. Protein sequences were manually curated and then aligned using the CLUSTAL-Wtool provided by MEGA X software (24). Phylogeny was performed using the same software. The trees were calculated using the rooted Neighbor-Joining (NJ) method (25) on distance matrices employing NEIGHBOR from the Clustal-X package. The Default *P*-distance method was used for distance analysis. *Rattus norvegicus* (rat) was used as the out-group. To generate a consensus tree, these trees were evaluated using the Clustalx software. The NJ Plot software application was used to plot a rooted tree. Because of the enormous number of sequences in the alignment, the bootstrapping of the “alignment dataset” was limited to 1,000 times. Sequences with more than 90% bootstrap support value were confirmed and categorized.

**Table 1:** Animal S.N. (En.) names, sequence ID, amino acids, and molecular weight of caseins in five dairy animals.

**Casein (gene)**	**Animal S.N. (En.) names**	**Sequence ID**	**Amino acids**	**M.W. k.Da**
alpha-S1 (*CSN1S1*)	*Camelus dromedaries* (Arabian camels)	NP_001290495.1	222	25.843
	*Ovis aries* (Sheep)	ACJ46472.1	214	24.315
	*Capra hircus* (Goats)	ALJ30147.1	213	24.130
	*Bos taurus* (Cattle)	1308122A	214	24.435
	*Bubalus bubalis* (Water buffalos)	APQ30583.1	214	24.312
	*Rattus norvegicus* (Rats)	NP_620229.2	284	31.801
alpha-S2 *(CSN1S2)*	*Camelus dromedaries* (Arabian camels)	NP_001290490.1	193	22.964
	*Ovis aries* (Sheep)	NP_001009363.1	223	26.331
	*Capra hircus* (Goats)	CAB94236.1	223	26.341
	*Bos taurus* (Cattle)	NP_776953.1	222	26.120
	*Bubalus bubalis* (Water buffalos)	ADW77639.1	222	26.223
	*Rattus norvegicus* (Rats)	NP_001099211.1	179	20.231
beta-casein *(CSN2)*	*Camelus dromedaries* (Arabian camels)	NP_001290492.1	232	26.217
	*Ovis aries* (Sheep)	QST88854.1	222	24.946
	*Capra hircus* (Goats)	AAK97639.1	223	24.992
	*Bos taurus* (Cattle)	AAB29137.1	224	25.098
	*Bubalus bubalis* (Water buffalos)	NP_001277808.1	224	25.101
	*Rattus norvegicus* (Rat)	NP_058816.2	231	25.345
kappa-casein *(CSN3)*	*Camelus dromedaries* (Arabian camels)	KAB1281948.1	219	24.717
	*Ovis aries*(Sheep)	AAP69943.1	162	17.989
	*Capra hircus* (Goats)	AAM12026.1	162	17.896
	*Bos taurus* (Cattle)	NP_776719.1	190	21.269
	*Bubalus bubalis* (Water buffalos)	NP_001277901.1	190	21.397
	*Rattus norvegicus* (Rats)	NP_113750.1	178	19.548

#### Multiple sequence alignment

For further analysis, combined alpha-S1-, alpha-S2-, beta-, and kappa-casein amino acid sequences in sheep, goats, cattle, Arabian camels, and water buffalos, respectively, were subjected to multiple sequence alignment using the Clustal Omegadatabase [CLUSTAL O (1.2.4)] (https://www.ebi.ac.uk/Tools/ msa/clustalo/), assisted by some manual adjustments to indicate the regions of similarity, identifying probably functional, structural, and evolutionary relationships between the sequences. A phylogenetic tree was rooted in a taxonomically distant organism (*Rattus norvegicus*).

#### Protein modeling

Genomic organization of chromosome six illustrating structure of casein-encoding genes *CSN1S1, CSN1S2, CSN2, CSN3*. The domains and phosphorylation sites associated with each protein subunit are presented from the NCBI databases. Protein 3D structures were predicted using the SWISS-MODEL homology modeling (swissmodel.expasy.org) method under the default parameters (26).

Three-dimensional representations of casein subunits were deduced from Kumosinski and Brown (27) for alpha-s1-casein and k-casein—Kumosinski, Brown (28) for beta-casein and Farrell Jr, Malin (29) for alpha-S2-casein. Protein structures exhibiting the highest homology were selected and developed as a template. Sequence homology for each sequence exceeded 70% and was thus considered highly reliable. Schematic illustrations were produced with the software package UCSF Chimera, candidate version 1.11.2.

### Computational chemical analysis of caseins

The chemical properties of caseins were *in silico*, computed by the ProtParam tool (https://web.expasy.org/protparam/), a tool of the ExPASy database that allows the computation of various physical and chemical features for a certain protein stored in Swiss-Prot or TrEMBL or for a user-entered protein sequence. The computed parameters include the molecular weight, instability index, hydrophobicity index, basic amino acids (%), negatively charged residues, and positively charged residues (30).

### Computational biological analysis of caseins

#### Prediction of antimicrobial activity of casein micelles

The publicly available AMPA tool (http://tcoffee.crg.cat/apps/ampa/do) was used to predict antimicrobial peptides. AMPA: an automated web server for the prediction of antimicrobial protein regions (31, 32). Whole-protein sequences were run in the AMPA using the default parameter values, i.e., a propensity threshold of 0.225 and a window size of 7 amino acids.

#### Prediction of antioxidant activity of caseins

The antioxidant activity index was calculated based on the casein content of hydrophobic amino acids, particularly tryptophan, methionine, isoleucine, leucine, and proline.

## Results

### Bioinformatics analysis of caseins

#### Phylogenetic analysis

**Figure 1:**
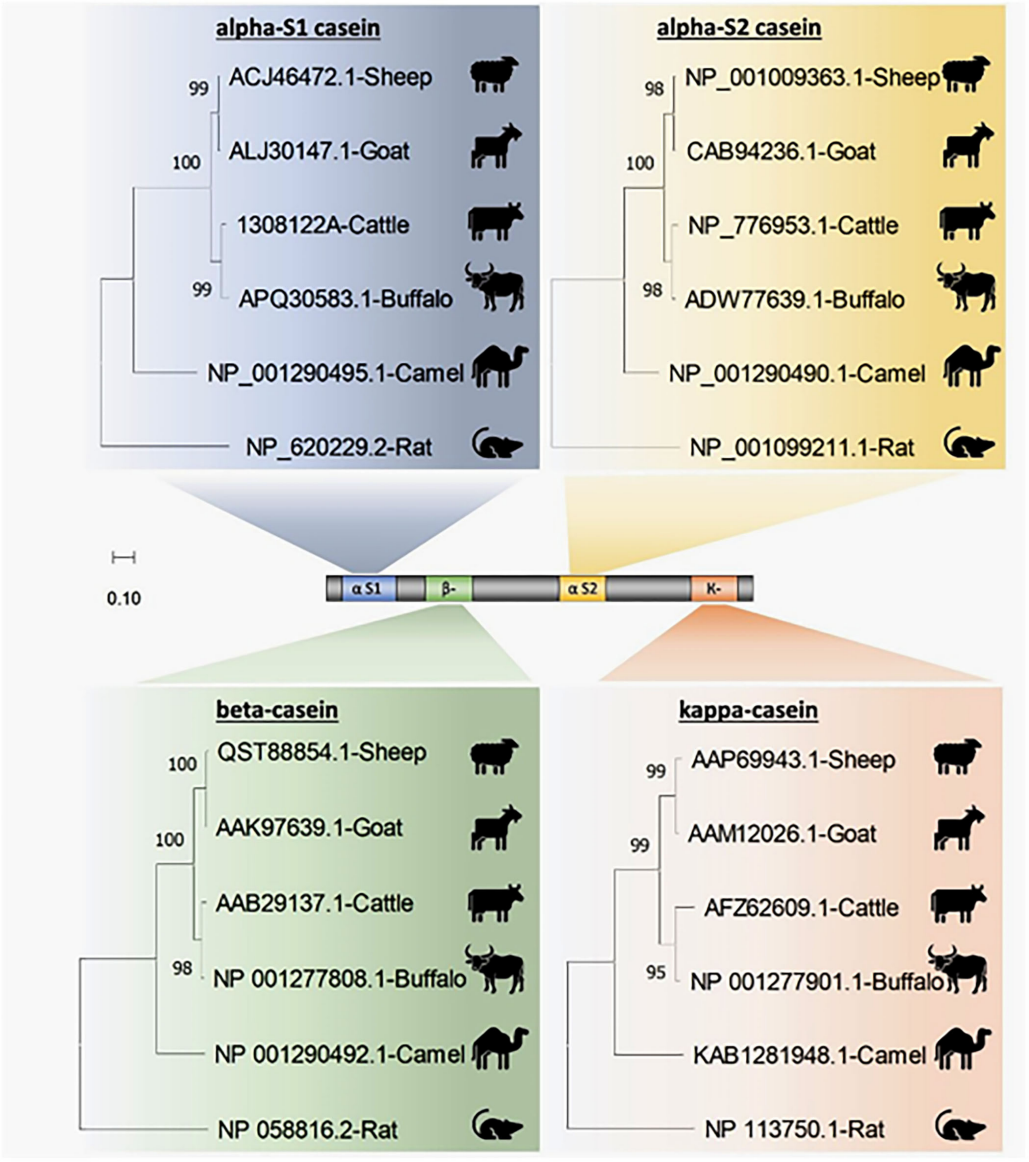
Phylogenetic analysis of casein subunits from different domestic animals. A neighbor-joining tree representing the phylogenetic relationship of sheep, goats, cattle, Arabian camels, and water buffalos was derived from the alignment of alpha-S1-, alpha-S2-, beta-, and kappa-casein amino acid sequences. The tree was rooted in *Rattus norvegicus*. Bootstrap support values are indicated on the nodes. Bootstrap values were calculated from 1,000 replications.

**Figure 2:**
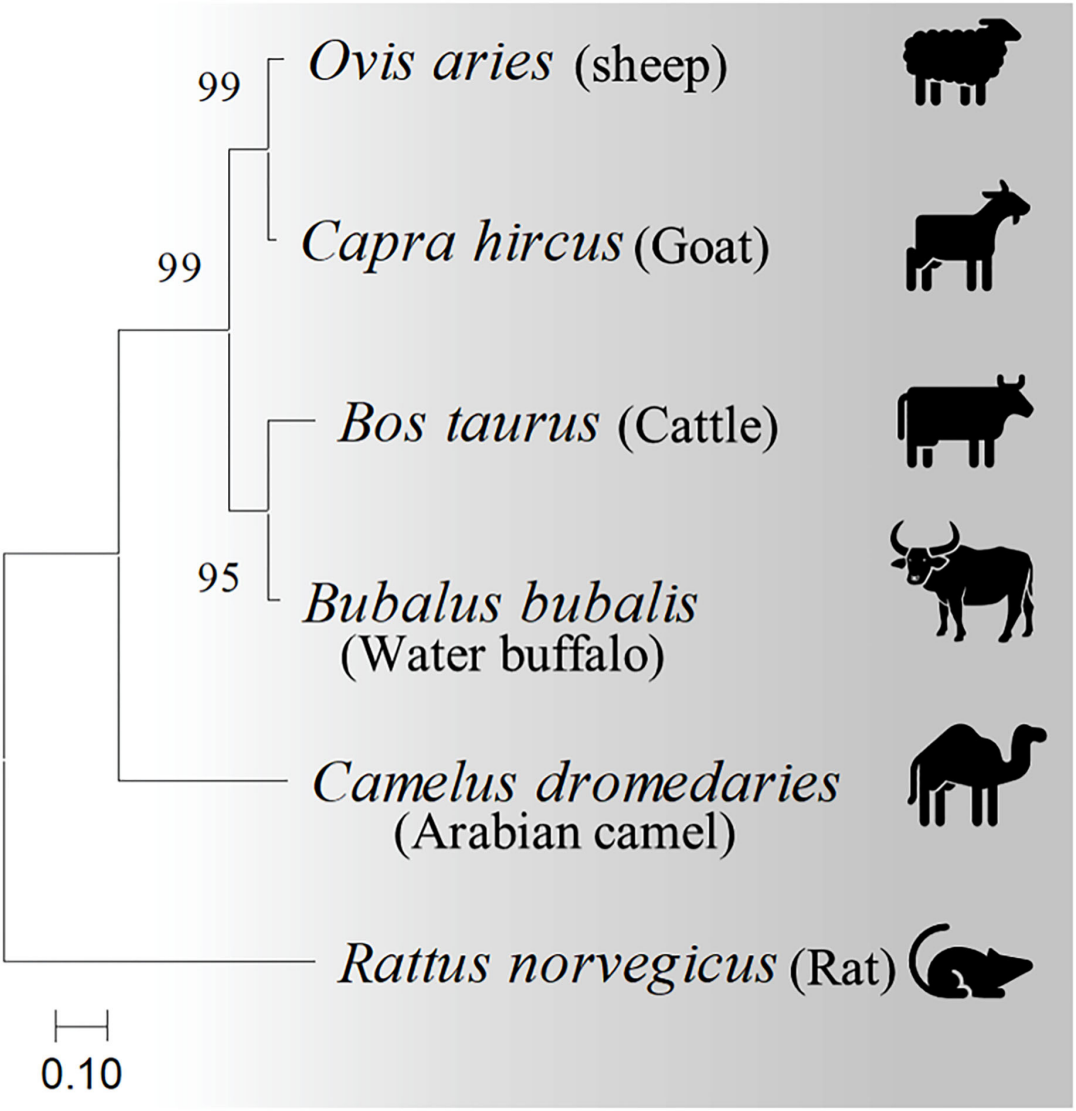
Phylogenetic relationships among casein homologs from different domestic animals. A neighbor-joining tree inferred from combined alpha-S1-, alpha-S2-, beta-, and kappa-casein amino acid sequences shows the phylogenetic positions of five other animals. The phylogenetic tree is derived from the alignment of four concatenated casein from sheep, goats, cattle, Arabian camels, and water buffalos. The tree was rooted in *Rattus norvegicus*. Bootstrap support values are indicated on the nodes. Bootstrap values were calculated from 1,000 replications.

Four casein sequences, alpha-S1, alpha-S2, beta, and kappa-casein, in five milk production animal species (Arabian camels, sheep, goats, cattle, and water buffalos) were individually (Figure 1) and in combination (Figure 2) analyzed by multiple sequence alignment for phylogeny study using the Clustal-X package and the rooted Neighbor-Joining (NJ) method (Supplementary Figure 1). This analysis was conducted to determine the phylogenetic relationship among sheep, goats, cattle, Arabian camels, and water buffalos through the alignment of caseins. The tree was rooted in a taxonomically distant organism (*Rattus norvegicus*). The phylogeny analysis revealed that the Arabian camel, which belongs to the Camelidae family, possesses low similarity with other domestic animals that belong to the Bovidae family, ranging between 60.55 with sheep to 62.22 with water buffalos (Table 2). Thus, the Arabian camel was sub-grouped individually (Figures 1, 2). The highest similarity (98.06) (Table 2) appeared between sheep and goats, which belong to the Bovidae family and are sub-grouped together by phylogeny analysis (Figures 1, 2). The phylogeny analysis revealed that cattle and water buffalo that belong to the Bovinae family possess a high similarity value (96.25) (Table 2), which included them in one subgroup (Figures 1, 2).

**Table 2:** Similarity values of the five domestic animals based on the combined caseins sequences.

**Organism**	**Arabian camels**	**Sheep**	**Goats**	**Cattle**	**Water buffalos**	**Rats**
Arabian camels	100					
Sheep	60.55	100				
Goats	60.77	**98.06**	100			
Cattle	61.23	87.27	87.16	100		
Water buffalos	62.22	88.20	88.10	**96.25**	100	
Rat	39.81	39.19	39.26	37.47	38.22	100

The bold values are the highest similarity values.

#### Protein modeling

**Figure 3:**
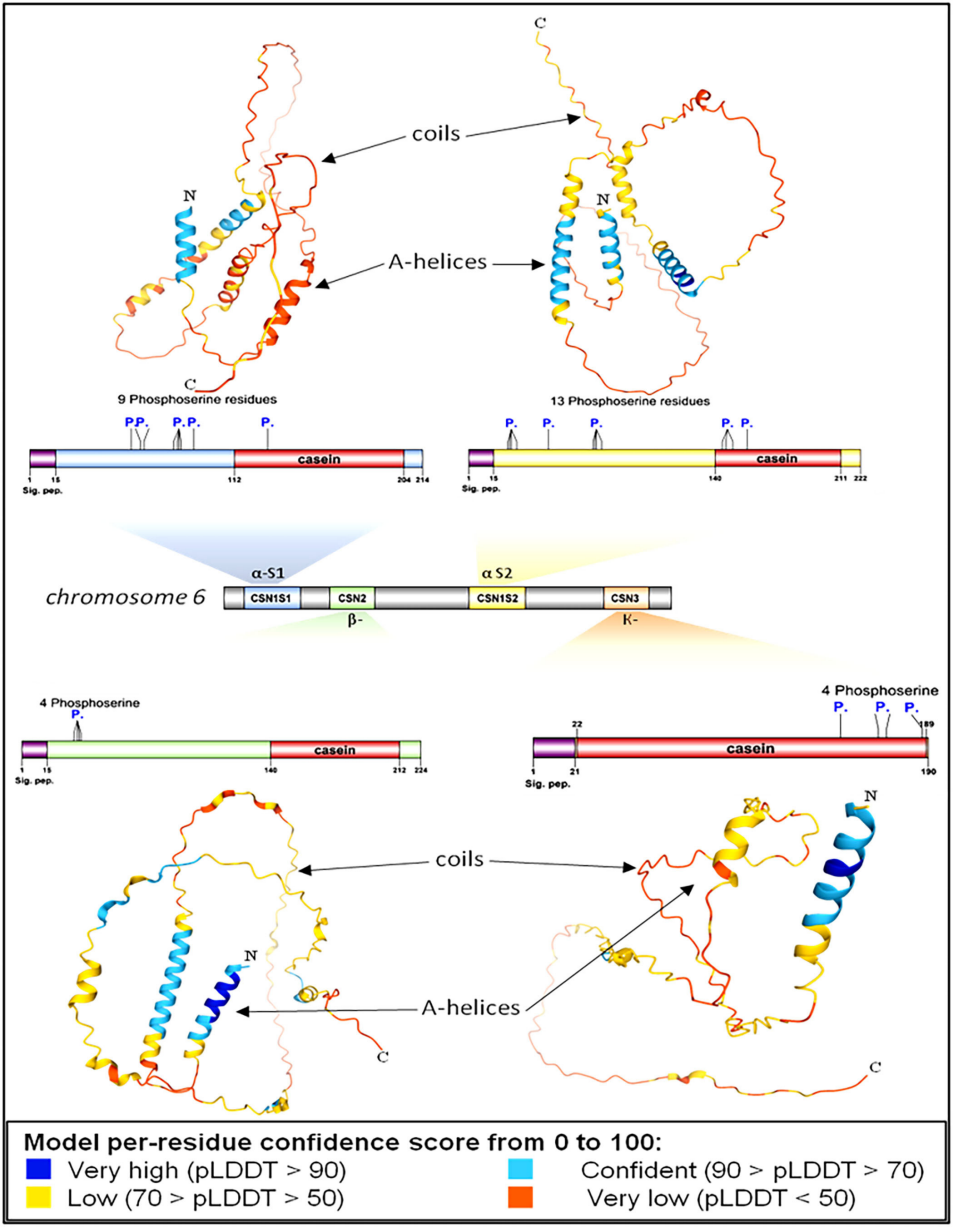
Genomic and structural characterization of milk caseins from *Bos taurus* (cattle). Casein subunit organization within chromosome number 6 (middle panel). The characteristics and genetic features of each casein subunit are detailed. Signal peptide domains (sig. pep.) and phosphorylation sites (P.) are indicated on each protein diagram. Ribbon diagrams illustrating the high-resolution structures of alpha-S1-, alpha-S2-, beta-, and kappa-casein. Proteins' N- and C-termini are indicated on protein models.

Protein modeling is considered a routine approach to provide structural models of proteins when no experimental structures are available. Protein modeling uses template protein structures to predict the conformations of other proteins with similar amino acids because small changes in the protein sequence usually result in small changes in the 3D structure. The 3D structure of caseins of cattle was used as a model for the rest of the domestic animals because it is the most popular and the most studied (Figure 3).

Protein modeling analysis revealed that the C-terminal ends had fewer secondary structures than the N-terminal of all caseins (Figure 3). In contrast to the C-terminal, the N-terminal exhibits a high degree of hydrophobicity that is not hydrolyzed in milk due to the presence of hydrophobic amino acid residues leucine and tryptophan in alpha S1; casein, proline, and tryptophan in alpha S2; casein, proline, and isoleucine in beta-casein; and alanine and valine in kappa casein. The analysis showed the presence of various numbers of phosphoserine residues in all caseins 9, 13, 4, and 4 in each alpha-S1-, alpha-S2-, beta-, and kappa-casein, respectively.

### Chemical features of caseins micelles

**Figure 4:**
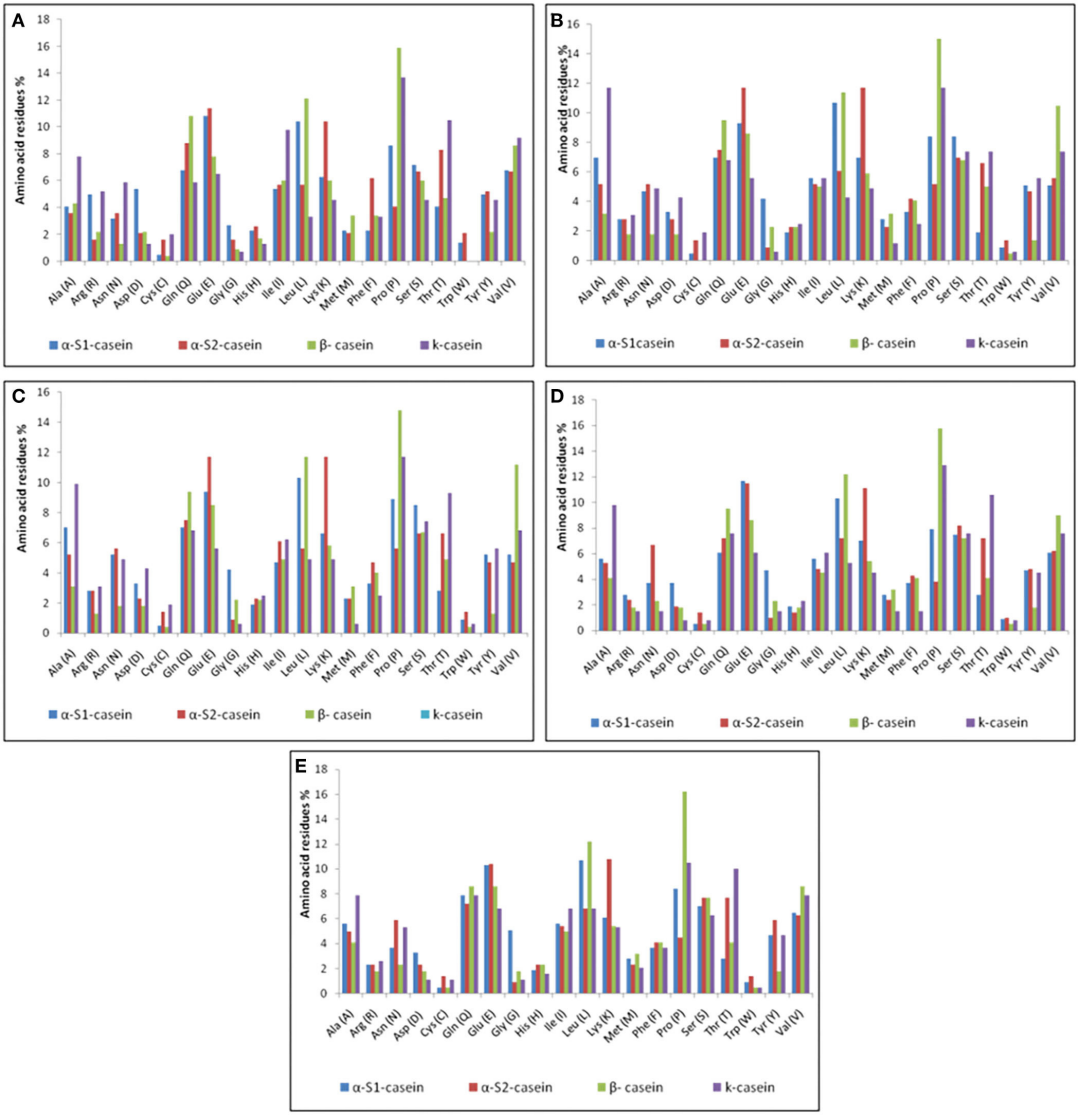
Amino acid compositions of caseins. **(A)** Arabian camel caseins. **(B)** Sheep caseins. **(C)** Goat caseins. **(D)** Cattle caseins. **(E)** Water buffalo caseins.

**Figure 5:**
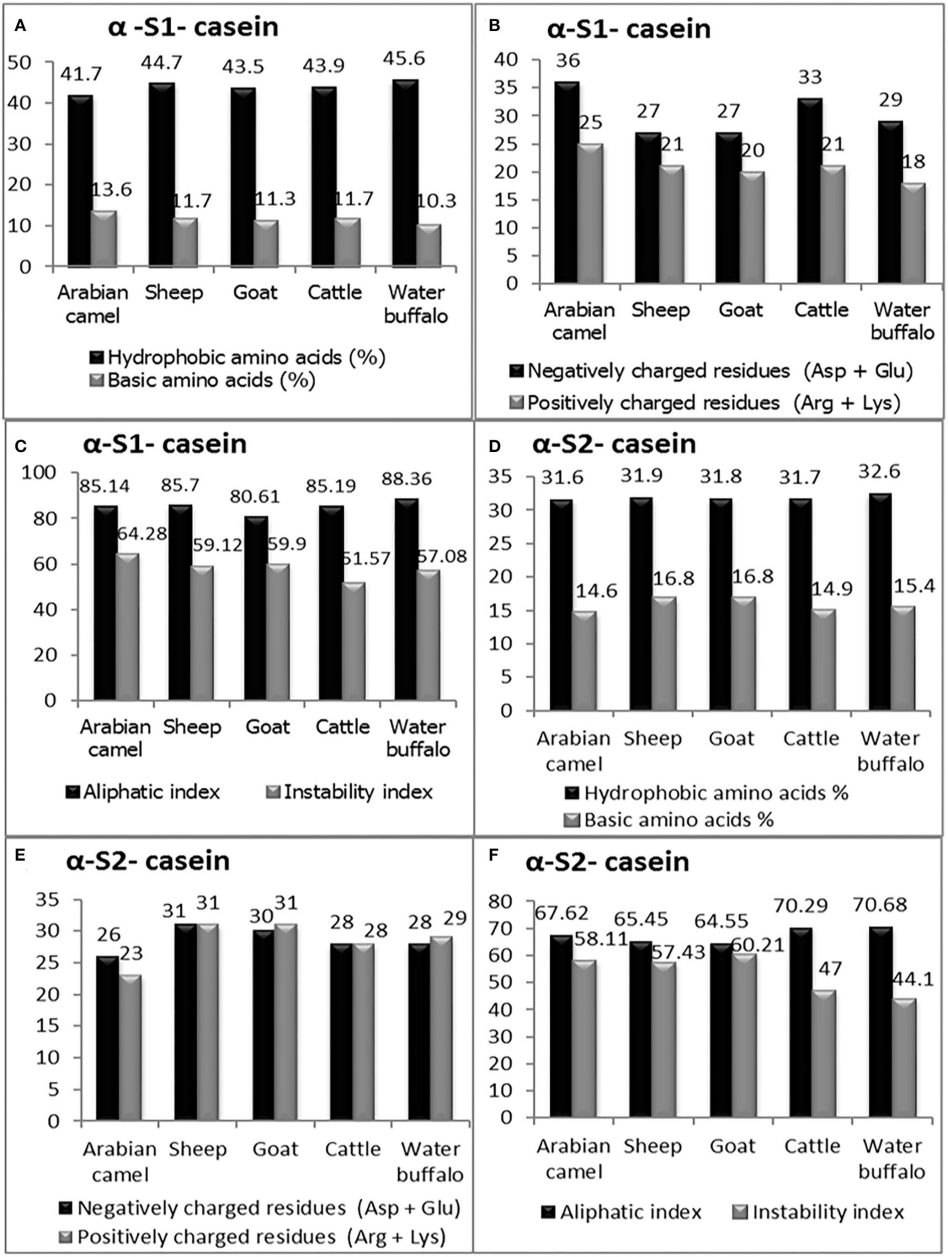
The computed parameters of α-S1-casein; **(A)** hydrophobic amino acids (%), basic amino acids (%). **(B)** Negatively charged residues (Asp + Glu), positively charged residues (Arg + Lys). **(C)** Aliphatic and instability indexes. The computed parameters of α-S2-casein **(D)** hydrophobic amino acids (%), and basic amino acids (%). **(E)** Negatively charged residues (Asp + Glu), positively charged residues (Arg + Lys). **(F)** Aliphatic and instability indexes.

**Figure 6:**
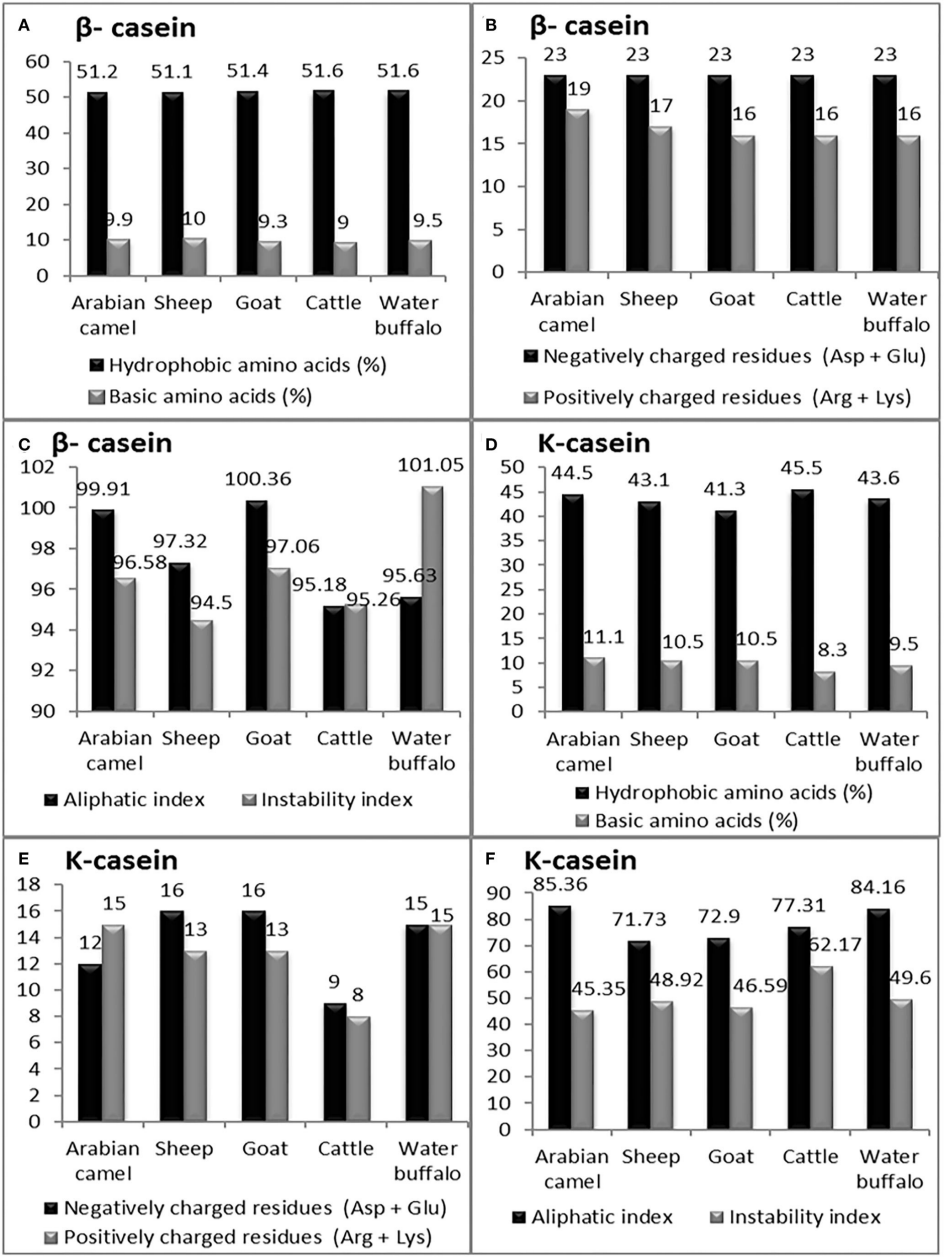
The computed parameters of β-casein; **(A)** Hydrophobic amino acids (%), Basic amino acids (%). **(B)** Negatively charged residues (Asp + Glu), positively charged residues (Arg + Lys). **(C)** Aliphatic and instability indexes. The computed parameters of K-casein. **(D)** Hydrophobic amino acids (%), Basic amino acids (%). **(E)** Negatively charged residues (Asp + Glu), positively charged residues (Arg + Lys). **(F)** Aliphatic and instability index.

Casein micelles (α_s1_-, α_s2_-, β-, and κ-casein) are present in all types of milk as self-assembled particles (33). The chemical structure of the casein micelles in milk production animals has not yet been sufficiently studied, except in cattle milk. The current *in silico* study comprehensively investigated the chemical characteristics of caseins as computed protein parameters; amino acid composition (Figure 4), protein size, molecular weight, hydrophobicity index (the percentage of hydrophobic amino acids), basic amino acid residues, negatively charged residues (Asp + Glu) and positively charged residues (Arg + Lys) in five milk production animals: Arabian camels, sheep, goats, cattle, and water buffalos (Figures 5, 6).

#### Alpha-S1-casein

The primary structure and percentage of each amino acid residue of alpha-S1-casein and alpha-S2-casein are presented in Table 3. The computed protein parameters indicated that the alpha-S1-casein of Arabian camels has an intermediate hydrophobicity index correlated with the presence of hydrophobic side chains in glycine, alanine, valine, leucine, isoleucine, proline, phenylalanine, methionine, and tryptophan, which ranged between 41.7% in Arabian camels and 45.6% in water buffalos, as well as the intermediate content of basic amino acid residues, which ranged between 10.3 and 13.6% in water buffalos and Arabian camels, respectively (Figure 5A). The analysis revealed that the Arabian camels and water buffalos showed the highest and lowest content of negatively charged residues (Asp + Glu) and positively charged residues (Arg + Lys) (Figure 5B). The alpha-S1-casein in Arabian camels showed the highest instability index (64.28), while the alpha-S1-casein in cattle was the lowest (51.57). On the other hand, the highest aliphatic side chains (aliphatic index), which are due to the presence of alanine, valine, isoleucine, and leucine residues, was 88.36 in water buffalos, while the lowest was 80.61 in goats—alpha-S1-casein (Figure 5C).

**Table 3:** Bactericidal propensity values (PV) for amino acid residues.

**Residue**	**PV**	**Residue**	**PV**	**Residue**	**PV**	**Residue**	**PV**
R	0.106	I	0.198	F	0.246	S	0.281
K	0.111	V	0.200	L	0.246	A	0.307
C	0.165	H	0.202	Q	0.248	P	0.327
W	0.172	N	0.240	G	0.265	E	0.449
Y	0.185	T	0.242	M	0.265	D	0.479

^*^Calculations are based on the average half-maximal inhibitory concentration (IC_50_), as detailed in the Methods section.

#### Alpha-S2-casein

The computed protein parameters indicated that the alpha–S2-casein of Arabian camels had a low hydrophobicity index, ranging between 31.60 in Arabian camels to 32.60 in water buffalos; the content of basic amino acid residues was the highest among the four caseins, ranging between 14.60 in Arabian camels to 16.80 in both sheep and goats (Figure 5D). The analysis revealed that sheep and Arabian camels showed the highest and lowest content of negatively charged residues and positively charged residues (Figure 5E). The alpha–S2-casein in goats showed the highest instability index (60.21), while the alpha–S2-casein in water buffalos presented the lowest (44.10); the highest aliphatic index was 70.68 in water buffalos; however, the lowest was 64.55 in goats—alpha–S2-casein (Figure 5F).

#### β-casein

The presence of hydrophobic domains in β-casein is featured; β-casein is the most hydrophobic casein and contains more prolyl residues than any other casein micelles. Hence, a molecular structure dominated by hydrophobic interactions of its surface than the α-S1, α-S2and k-casein would be expected. These characteristics appear to be manifested in the physicochemical properties of this protein. The hydrophobicity index of β-casein ranged between 51.10 in sheep and 51.60 in both cattle and water buffalos; unlike the hydrophobicity index, β-casein has the lowest basic amino acid content among the four caseins, ranging between nine in cattle to ten in sheep (Figure 6A). The analysis revealed that β-casein has the same content of negatively charged residues in all examined animals, while the positively charged residues ranged from 16 in goats, cattle, and water buffalos to 19 in Arabian camels (Figure 6B). The β-casein in goats exhibited the lowest instability index (94.50), whereas buffalos presented the highest (101.05). The highest aliphatic index was 100.36 in goats, while the lowest was 95.18 in cattle β-casein (Figure 6C).

#### K-casein

The computed analysis revealed that K-casein is less hydrophobic than β-casein and has a lower frequency of prolyl residues. The analysis showed that the hydrophobicity index of K-casein ranged from 41.30 in goats to 45.50 in cattle, and β-casein has the lowest basic amino acid content (9.5) in water buffalos to 11.10 in Arabian camels (Figure 6D). The analysis revealed that k-casein has a low content of negatively and positively charged residues in all examined animals. The negatively charged residues ranged from 9 in cattle to 16 in sheep and goats, while the positively charged residues ranged from 8 in cattle to 15 in Arabian camels (Figure 6E). The k-casein in Arabian camels showed the lowest instability index (45.35), while cattle presented the highest (62.17). The highest aliphatic index was 84.16 in water buffalos, while the lowest was 71.73 in sheep β-casein (Figure 6F).

### *In silico* biological features of caseins

#### Antimicrobial activity of caseins

*In silico*, antimicrobial index and bactericidal stretches were calculated for caseins α_s1_-, α_s2_-, β-, and κ-casein in the five animals understudy to discover the antimicrobial patterns of milk casein. The analysis showed that all caseins have hydrophobicity indexes (Table 4), which enable milk casein to hydrophobically interact with pathogens with the consideration that showed the highest hydrophobicity index. The analysis also revealed that α-S2-casein and β-casein showed the highest and the lowest content of basic amino acids, respectively (Table 4), while α-S1-casein and k-casein showed intermediate content of basic amino acids, which means that all caseins can have bacteriostatic interactions with microorganisms. The more interesting result was that α-S2-casein showed the highest number of bactericidal stretches (three in Arabian camels, one in each sheep and goats, three in cattle, and two in water buffalos) compared to only one bactericidal stretch in Arabian camel β-casein and Arabian camel k-casein (Table 4). These results gave preference to Arabian camel casein due to containing 3, 1, and 1 bactericidal stretches in each of α-S2-casein, β-casein, and k-casein, respectively.

**Table 4:** The computed biological parameters of caseins in five milk-producing animals.

**Casein type**	**Organism Name**	**Hydrophobicity index**	**Basic amino acids (%)**	**Bactericidal stretches**	**Average antimicrobial index**	**Antioxidant activity index**
α -S1-casein	Arabian camels	41.7	13.6	0	0.267	26.7
	Sheep	44.7	11.7	0	0.264	27.5
	Goats	43.5	11.3	0	0.267	26.2
	Cattle	43.9	11.7	0	0.267	26.6
	Water buffalos	45.6	10.3	0	0.267	27.5
α-S2-casein	Arabian camels	31.6	14.6	3	0.253	17.6
	Sheep	31.9	16.8	1	0.254	18.8
	Goats	31.8	16.8	1	0.253	19.6
	Cattle	31.7	14.9	3	0.251	18.2
	Water buffalos	32.6	15.4	2	0.252	19
β-casein	Arabian camels	51.2	9.9	1	0.265	37.4
	Sheep	51.1	10	0	0.265	34.6
	Goats	51.4	9.3	0	0.265	34.5
	Cattle	51.6	9	0	0.267	35.7
	Water buffalos	51.6	9.5	0	0.267	36.6
K-casein	Arabian camels	44.5	11.1	1	0.247	26
	Sheep	43.1	10.5	0	0.263	22.8
	Goats	41.3	10.5	0	0.261	23.4
	Cattle	45.5	8.3	0	0.260	25.8
	Water buffalos	43.6	9.5	0	0.255	26.2

**Figure 7:**
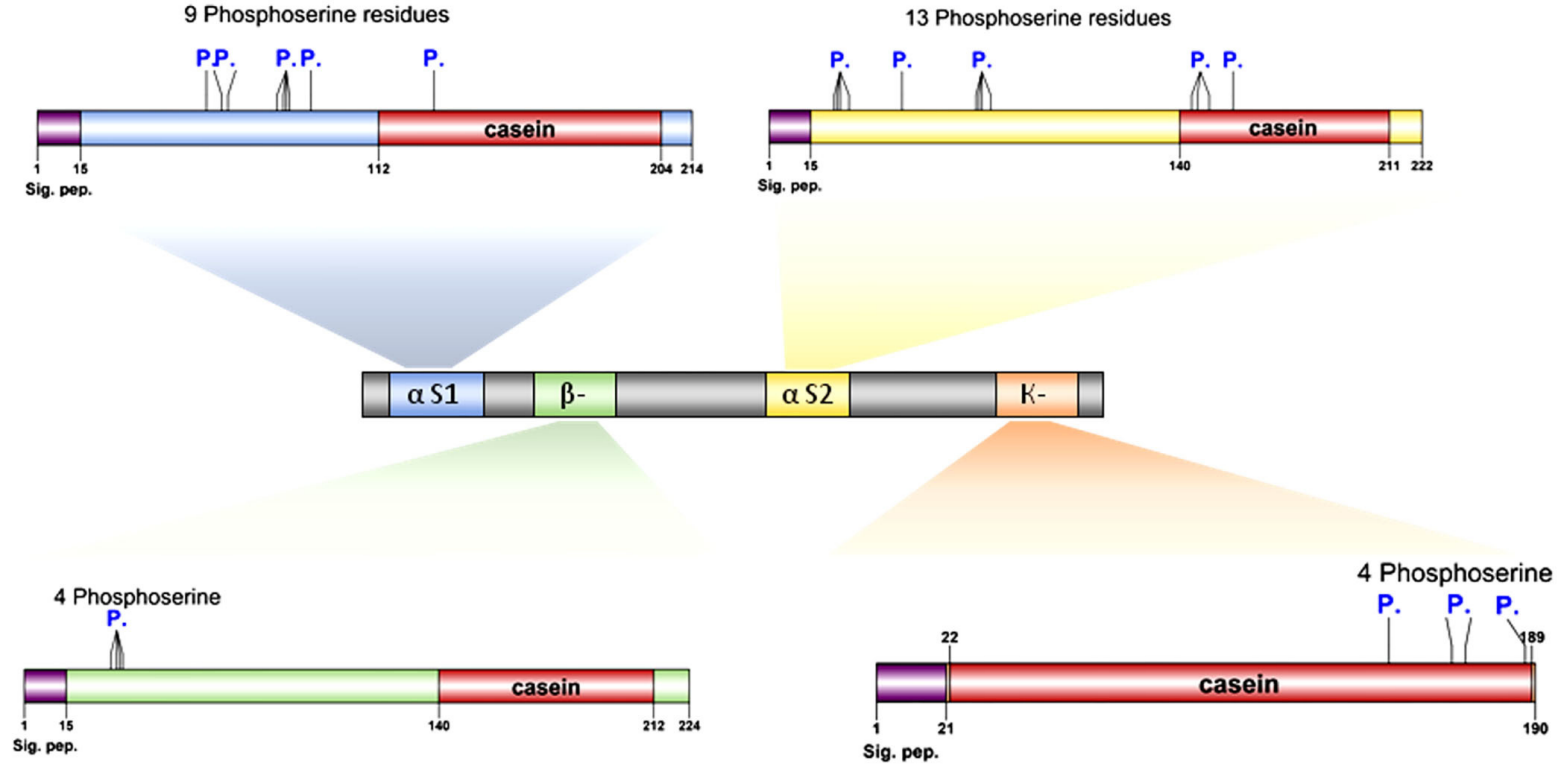
Proteomic and structural characterization of milk caseins from Bos taurus (cattle). Signal peptide domains (sig. pep.) and phosphorylation sites (P.) are indicated on each protein diagram.

The presence of phosphorylated amino acids dramatically affects the antimicrobial activity of the caseins. The analysis of cattle caseins as the most popular and most studied model among animals showed the presence of phosphoserine residues in all casein micelles. The α-S2-casein contained the largest number of phosphoserine residues (13), followed by α-S1-casein (9), while each β-casein and k-caseins contained four phosphoserine residues (Figure 7).

#### Antioxidant activity of caseins

The antioxidant activity index is attributed to the interesting antioxidant properties of the protein due to its content of hydrophobic amino acids, particularly tryptophan, methionine, isoleucine, leucine, and proline. The computed analysis revealed the hydrophobic amino-acid content of caseins α_s1_-, α_s2_-, β-, and κ-casein. The analysis showed that β-casein possessed the highest antioxidant activity index, ranging between 34.5 in goats and 37.4 in Arabian camels (Table 4). Both alpha-S1-casein and k-casein possessed an intermediate antioxidant activity index, whereas the alpha -S1-casein ranged between 26.2 in goats to 27.5 in both sheep and water buffalos (Table 4), while the K-casein ranged between 22.8 in sheep to 26.2 in water buffalos. The alpha-S2-casein possessed the lowest antioxidant activity index, ranging between 17.6 in Arabian camels and 19.6 in goats (Table 4).

## Discussion

Phylogenetic comparative analysis is crucial in determining phylogenetic relationships between two or more genomes to discover the differences and similarities among organisms (34–41).

The phylogeny analysis divided the five domestic animals according to combined and caseins into three subgroups, which were completely consistent with the genetic background of the species. The observed data assumed that the five domestic animals had evolved following a close evolutionary model. Interestingly, the result of our analysis divided the domestic animals based on caseins into three subgroups; one consisted of only one species (Arabian camels), the second contained both cattle and water buffalos, and the third included sheep and goats. This result could be explained by the zoological taxa of these animals. The Arabian camel belongs to the Camelidae family; the Bovidae family comprises cows, buffalos, sheep, and goats, and the Bovinae family consists of both cows and buffalos.

The positions of the Arabian camel in all phylogenetic tree topologies are the same. In the same context, the rest of all species topologies are mostly the same, whether in the tree constructed based on the combined caseins or in the trees constructed based on the individual caseins. The genetic distances and common ancestors, either between sheep and goats or cattle and water buffalos in all trees, are quite similar.

The 3D modeling of casein molecules identifies regions with different levels of organization within each casein type that affect the biological functions of the protein. When purified caseins were utilized as substrates, the proteinases were efficiently hydrolyzed at the sites with the least secondary structure. The C-terminal ends of all casein sequences are highly unstructured compared to their N-terminal ends, which are well-structured. The phosphorylated sites of the casein were poorly hydrolyzed, which is in agreement with previous studies (42–44).

Divergence in casein genes and the presence or absence of one or more genes encoding caseins explain the different biological and chemical functions of the casein micelles (45). The genes encoding α-caseins are absent in most mammalian species; for example, human milk lacks αs2-casein, and it may be possible that its role in casein micelle formation could be shifted to αs1-casein (14). In contrast, genes encoding β-and κ-caseins are widely distributed among mammals.

Amino acid composition and the type of protein residue sequence determine the structure, hydrophobicity, biological functions, and nutritional value of milk casein (46). Milk casein displays antimicrobial activity by preventing pathogen adhesion and invasion by either directly interacting with the pathogen or modifying the host environment, resulting in microorganism growth inhibition (47, 48).

The antimicrobial activity of caseins against pathogens is specific due to their affinity for polarized bacterial membranes rather than the depolarized membranes of eukaryotic cells (49). There is mounting evidence suggesting that the antibacterial properties of milk hydrolysates are linked to the production of peptides with α-helical structures. The antibacterial action is greatly affected by the presence of phosphorylated residues of certain amino acids or chemical changes in C- and N- termini (49, 50). Antimicrobial peptides can also kill bacteria by aggregating in the cytoplasmic membrane, altering their membrane permeability and triggering cell death (51, 52).

Peptides and proteins' antibacterial activity could be related to their net charge or hydrophobic characteristics. Because the majority of antibacterial peptides are positively charged, they electrostatically bond to the negatively charged components of the bacterial cell wall, potentially causing cell wall disintegration (53, 54).

Our *in silico* analysis indicated the antimicrobial characteristics of caseins based on the hydrophobicity, basic amino acid content, negatively charged residues (Asp + Glu), and positively charged residues (Arg + Lys) were consistent with the results of several studies (19, 55–63). Because α-S2 casein is not found in human milk, hydrolysates of bovine α-S2 casein have shown promise as a novel microbiota-modifying agent in the human gastrointestinal tract (56). Strömqvist and Falk (57) found that *Helicobacter pylori*, an early-life pathogen, is prevented from adhering to human stomach mucosa by the small subunit of k-casein.

Our *in silico* analysis gave the lion's share to the casein of the Arabian camel, which is consistent with the *in vitro* results of several studies (19, 64, 65), indicating the value of bioinformatics tools besides the recent genome editing approaches (66, 67) to accurate prediction and determination of antimicrobial features of casein micelles.

The antioxidant activity index presented in Table 4 revealed that all caseins may possess antioxidant activity levels. The β-casein exhibited greater antioxidant activity, particularly Arabian camel β- casein. These results were in agreement with the research by Salami, and Moosavi-Movahedi [68], who reported that camel β-casein presented higher antioxidant activity when compared to the camel's other caseins. These researchers attributed camel casein's intriguing antioxidant characteristics to the protein's highest hydrophobicity index and its main sequence, which play a key role in free radical scavenging. This is due to the greater antioxidant amino-acid content, such as Tyr, Met, Ile, Leu, and Pro of camel casein, compared to other caseins. Proteins' antioxidant capabilities are influenced not only by their amino-acid residue composition but also by their location and accessibility (68).

## Conclusion

This study is a step toward a better understanding of the great importance and utility of bioinformatics tools in the accurate prediction, comprehensive characterization, and *in silico* determination of the biological activities of milk proteins. This study determined the relationship between five domestic dairy animals based on phylogenetic analysis of caseins. The results enhanced our knowledge of casein micelles, their chemical features, and potential biological functions. Therefore, it is likely that the presence of such features in the milk structure could have additional beneficial health effects, such as reduction of oxidative stress in the stomach and antimicrobial agents against harmful pathogens.

## Data availability statement

The raw data supporting the conclusions of this article will be made available by the authors, without undue reservation.

## Author contributions

AH, MA, and AE: conceptualization and investigation. AH and OA: methodology. AH, AA, and OA: software. AH, AO, and SA: validation. AH, HE, and AO: formal analysis. ME-S, IA, HE, and OA: resources. OA, AM, AO, and AH: data curation. AH, HE, and SA: writing original draft preparation. AH and AE: writing, review, and editing. AH and MA: visualization and supervision. AA, MA, and IA: funding acquisition. All authors have read and agreed to the published version of the manuscript.

## Conflict of interest

The authors declare that the research was conducted in the absence of any commercial or financial relationships that could be construed as a potential conflict of interest.

## Publisher's note

All claims expressed in this article are solely those of the authors and do not necessarily represent those of their affiliated organizations, or those of the publisher, the editors and the reviewers. Any product that may be evaluated in this article, or claim that may be made by its manufacturer, is not guaranteed or endorsed by the publisher.

## Abbreviations

NCBI: National Center for Biotechnology Information
NJ: Neighbor-Joining
*CSN1S1*: Casein Alpha S1
*CSN1S2*: Casein Alpha S2
*CSN2*: Casein Beta
*CSN3*: Casein Kappa
IC_50_: inhibitory concentration
PV: propensity value
3D: Three-dimensional
C-terminal: carboxyl-terminus or carboxy-terminus
N-terminal: amino-terminus or NH2-terminus.
