# Supplementary material for: Phylogenetic comparative analysis: Chemical and biological features of caseins (alpha-S-1, alpha-S-2, beta- and kappa-) in domestic dairy animals
Source: Front Vet Sci. 2022 Sep 15;9:952319. doi: 10.3389/fvets.2022.952319 (PMC9519386; doi:10.3389/fvets.2022.952319)
Supplement: Supplementary file 1 [file Table_1.DOCX]

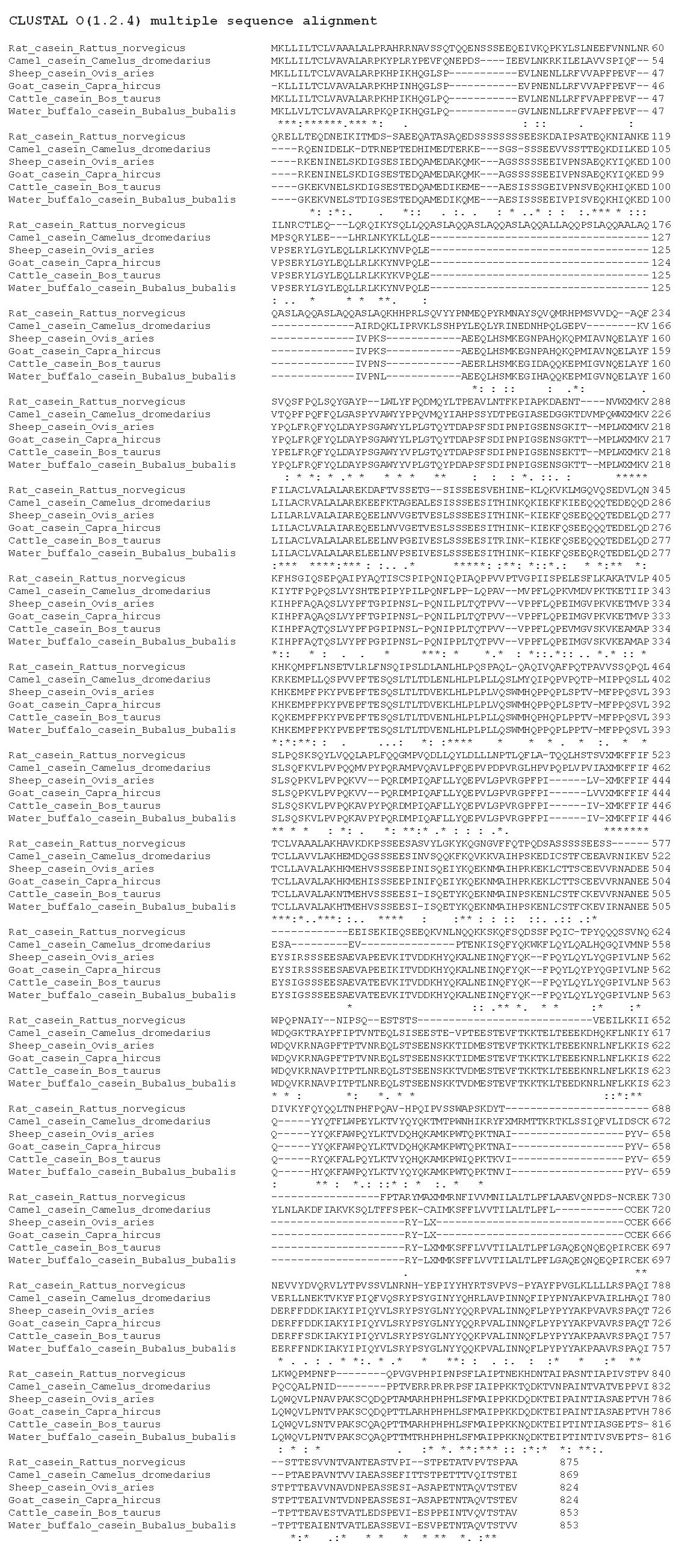


**Figure S 1**: Multiple sequence alignment of combined alpha-S1-, alpha-S2-, beta-, and kappa-casein amino acid sequences in sheep, goat, cattle, Arabian camel and water buffalo rooted to a taxonomically distant organism (*Rattus norvegicus*).
